# Microscopic and Molecular Characterization of the Prehaustorial Resistance against Wheat Leaf Rust (*Puccinia triticina*) in Einkorn (*Triticum monococcum*)

**DOI:** 10.3389/fpls.2016.01668

**Published:** 2016-11-09

**Authors:** Albrecht Serfling, Sven E. Templer, Peter Winter, Frank Ordon

**Affiliations:** ^1^Institute for Resistance Research and Stress Tolerance, Julius Kuehn-Institute, Federal Research Centre for Cultivated PlantsQuedlinburg, Germany; ^2^Interdisciplinary Center for Crop Plant Research, Martin Luther University Halle-WittenbergHalle, Germany; ^3^Department of Plant Developmental Biology, Max Planck Institute for Plant Breeding ResearchCologne, Germany; ^4^GenXpro GmbHFrankfurt am Main, Germany

**Keywords:** Einkorn, leaf rust, non-host, plant defense, prehaustorial resistance, transcriptomics

## Abstract

*Puccinia triticina* f. sp. *tritici* (Eriks.), the causal agent of leaf rust, causes substantial yield losses in wheat production. In wheat many major leaf rust resistance genes have been overcome by virulent races. In contrast, the prehaustorial resistance (phr) against wheat leaf rust detected in the diploid wheat Einkorn (*Triticum monoccocum* var. *monococcum*) accession PI272560 confers race-independent resistance against isolates virulent on accessions harboring resistance genes located on the A-genome of *Triticum aestivum*. Phr in PI272560 leads to abortion of fungal development during the formation of haustorial mother cells and to increased hydrogen peroxide concentration in comparison to the susceptible accession 36554 (*Triticum boeoticum* ssp. *thaoudar* var. *reuteri*). Increased peroxidase and endochitinase activity was detected in PI272560 within 6 h after inoculation (hai). Comparative transcriptome profiling using Massive Analysis of cDNA Ends (MACE) in infected and non-infected leaves detected 14220 differentially expressed tags in PI272560 and 15472 in accession 36554. Of these 2908 and 3004, respectively, could be assigned to Gene Ontology (GO) categories of which 463 were detected in both accessions and 311 were differentially expressed between the accessions. In accordance with the concept of non-host resistance in PI272560, genes with similarity to peroxidases, chitinases, β-1,3-glucanases and other pathogenesis-related genes were up-regulated within the first 8 hai, whereas up-regulation of such genes was delayed in 36554. Moreover, a Phosphoribulokinase gene contributing to non-host resistance in rice against stripe rust was exclusively expressed in the resistant accession PI272560. Gene expression underpinned physiological and phenotypic observations at the site of infection and are in accordance with the concept of non-host resistance.

## Introduction

Leaf rust caused by *Puccinia triticina* (Eriks) is an important fungal disease and the most common rust of wheat (*Triticum aestivum*) causing high yield losses up to 60% worldwide (Sayre et al., 1998). To combat leaf rust epidemics the integration of resistances in cultivars is environmental friendly and cost saving (Dubin and Brennan, 2009). Single leaf rust resistance genes (*Lr*-genes) have been widely deployed in wheat cultivars, already. However, most of the more than 70 known *Lr*-genes mainly effective at the seedling stage confer race-specific resistance to *P. triticina* isolates in wheat and *Lr*-genes, e.g., *Lr3, Lr10, Lr13, Lr17b, Lr26* and *Lr37* have been overcome meanwhile (Hysing et al., 2006; Serfling et al., 2011; Kolmer, 2013). Kilpatrick (1975) estimated that the average time a single resistance gene against leaf rust in wheat is efficient, is about 5–6 years while the incorporation of new and efficient *Lr*-genes into wheat cultivars needs approximately 10–15 years ^1^. Hence to prevent epidemics after a breakdown of single resistance genes, expensive and potentially eco-toxic fungicide treatments are conducted (Frampton et al., 2006; Komarek et al., 2010) which may result in the emergence of fungicide- tolerant or resistant fungal strains (Cook, 2001). Therefore, adult plant resistances (APR), e.g., *Lr34* which in contrast to single resistance genes confers non-race-specific resistance also against powdery mildew (*Blumeria graminis*) and stem rust (*Puccinia graminis*) has been introduced from a landrace into wheat cultivars (Spielmeyer et al., 2013). However, cultivars carrying this *Lr-*gene show a reduced number of uredospore pustules at best. Whereas at least some of the race specific resistance genes like *Lr1, Lr10* and *Lr21* display features of classical resistance genes, i.e., coiled coil (CC), nucleotide-binding-site (NBS), and leucine-rich-repeat (LRR) motifs (Feuillet et al., 2003; Huang et al., 2003; Cloutier et al., 2007). *Lr34* turned out to encode a putative ATP binding transporter protein leading to a quantitative race-non specific resistance (Krattinger et al., 2009). Similar to quantitative resistance conferred by *Lr34*, prehaustorial resistance (phr) most likely confers race non specific resistance to *P. triticina*, but in the majority of cases phr turned out to be not associated with macroscopic visible necrosis (Anker, 2001 unpublished; Anker and Niks, 2001) in *T. monococcum* accessions. Niks (1988) postulated that non-host resistance to rust and powdery mildew appears to be usually prehaustorial and after the observation of more than 50% of early aborted leaf rust infection units, Anker and Niks (2001) assumed a non-host resistance in a few *T. monococcum* accessions while Rubiales and Niks (1995) supposed a similar mode of action as for *Lr34* and *Lr46*. However, there are indications that the molecular basis of *Lr46* differs from *Lr34* (Lagudah, 2011). Non-host rust interactions have been investigated between several cereal rust species and *Brachypodium* spp., barley (*Hordeum vulgare*) and rice (*Oryza sativa* ssp. *japonica* and *indica*). In these studies a wide range of interactions from basic incompatibility and phr to the generation of pustules reduced in size (reviewed by Bettgenhaeuser et al., 2014; Dracatos et al., 2014) were observed. The molecular mechanisms underlying phr against *P. triticina* are still not known in detail. Non-host resistance is characterized by the increased expression of pathogenesis-related genes (*Pr*-genes) and the production of reactive oxygen species (ROS) after the recognition of general elicitors from pathogens in a non specific manner (Heath, 2000; Mysore and Ryu, 2004). With regard to phr, no information on differentially expressed genes leading to efficient (prehaustorial) resistance is available up to now. In this respect genome wide transcription profiling with, e.g., Massive Analysis of cDNA Ends (MACE, Kahl et al., 2012; Zawada et al., 2014; Nold-Petry et al., 2015) is well suited to detect differences in gene expression between *T. monococcum* accessions with different levels of resistance against leaf rust. The comparison of differentially expressed genes within the first 24 hai comprises the time after the germination of uredospores up to the beginning of the formation of the first haustoria within mesophyll cells of susceptible plants (Bolton et al., 2008). To investigate the molecular processes accompanying fungal invasion, next generation sequencing (NGS) by RNA-seq has been successfully employed, e.g., in the pathosystems *Populus trichocarpa* – *Melampsora larici-populina* (Petre et al., 2012), or *Glycine max* and *Phakopsora pachyrhizi* (Tremblay et al., 2011). Compared to RNAseq, where the number of sequences obtained from a particular cDNA depends on the abundance and the size of the respective cDNA, MACE generates only a single tag from each cDNA. The tag is obtained from 300 to 800 bp from the 3′-end. Therefore, each cDNA is counted only once irrespective of its size. Consequently, much less sequences – resulting in lower costs – are required to obtain the same quantitative accuracy as RNA-seq. Moreover, the TrueQuant technology embedded in MACE ensures that the resulting quantitative data are free of a PCR bias (Kahl et al., 2012; Zawada et al., 2014; Nold-Petry et al., 2015). In order to get detailed information on the phr to *P. Triticina*, the following studies have been conducted (i), leaves of a *T. monococcum* accession showing phr and a susceptible *T. boeoticum* accession were inoculated with *P. triticina* isolates with different virulence patterns, (ii) these accessions were microscopically analyzed to detect the inhibition of fungal growth, phenolic compounds, hydrogen peroxide and reduced fluorescence of fungal cell walls by endochitinase activity, (iii) genome-wide transcription profiling of mRNA from leaves of resistant and susceptible accessions harvested within the first 24 h after infection was applied using MACE in order to detect differentially expressed genes, (iv) respective genes were assigned to Gene Ontology (GO) categories enabling a deeper insight into compatible and incompatible resistance reactions and explaining a large deal of the mechanisms underlying non-host resistance against leaf rust.

## Materials and Methods

### Plant Material and Growing Conditions

For all experiments, seeds of the resistant *T. monococcum* and the susceptible *Triticum boeoticum* accessions, i.e., resistant PI272560 (*T*. *monococcum* var. *monococcum* variety “Ungarn white,” Anker and Niks, 2001) and susceptible accession 36554 (*T. boeoticum* ssp. *thaoudar* var. *reuteri*, variety “Angora,” Anker and Niks, 2001) were obtained from the gene bank of the Leibniz Institute of Plant Genetics and Crop Plant Research (IPK, Gatersleben, Germany) and the National Plant Germplasm System (NPGS) of the United States Department of Agriculture (Aberdeen, ID, USA). Seeds were germinated on moist filter paper in petri dishes and 3 days after germination three plantlets each were transferred to three pots with a size of 11 cm × 11 cm (height and width), filled with soil (Archut- Fruhstorfer Erde, HAWITA, Oldenburg Germany). Cultivation was conducted at 80% ± 10% humidity, at 20°C ± 2°C and a light intensity higher than 300 ± 15 μmol under daylight conditions (16 h) on the level of the soil surface.

### Tests for the Presence of Known Resistance Genes Located on the A-Genome

Each pot, i.e., three replications per Thatcher NIL and *T. monococcum* and *T. boeoticum* accession, was inoculated with 2 mg of leaf rust uredospores mixed with 2 mg of dry powdered clay 11 days after planting using a settling tower (Hoogkamp et al., 1998). The single spore isolates wxr77, isolate 167/176wxr, 13/20wxr and 58 wxr were kindly provided by Dr. Lind (Julius Kuehn-Institute, Quedlinburg, Germany) and are originated from a collection, cultivated firstly by Nover and Lehmann (1967). Furthermore, uredospores from leaves were collected in 2001 and 2004 from flag leaves of the cultivar Borenos (EC stage 60) on the experimental station of the JKI at Aschersleben (coordinates N 51.756541; E 11.431193). All single spore isolates were cultivated and multiplicated on leaves of the wheat variety Monopol. The resulting isolates Hk12/3-01 and Hk1/3-04 and the above mentioned isolates were used in pot trials under the above mentioned conditions for virulence/avirulene analysis on leaves of NILs of the spring wheat cultivar Thatcher (Thatcher-NILs) carrying *Lr*-genes located on the A genome. These are *Lr10* (Thatcher^∗^6/Exchange, Feuillet et al., 2003), *Lr11* (Thatcher^∗^6/Hussar, Soliman et al., 1964), *Lr17* (Thatcher^∗^6/Klein Lucero, Dyck and Kerber, 1977), *Lr20* (Thatcher^∗^6/Jimmer, Neu et al., 2002), *Lr28* (Thatcher^∗^6/C-77-1, Friebe et al., 1996), *Lr37* (Thatcher^∗^6/VPM, Bariana and McIntosh, 1993), and an additional variety which carries *Tm* (cultivar Ks92WGRC23, Hussien et al., 1997) and the susceptible standard cultivar Thatcher. Furthermore, these isolates were tested for virulence on leaves of the accessions PI272560 and 36554. Isolates were stored and spores were produced according to the method of Mebrate et al. (2006). Position of pots were randomized and changed daily to avoid possible effects of location.

### Inoculation of Plants for MACE-Analysis

In order to characterize the molecular mechanisms of phr to leaf rust and to differentiate between the resistant accession PI272560 and the susceptible accession 36554, these accessions were planted in 48 beaker glasses with a volume of 2 l for 14 days respectively under the above mentioned greenhouse conditions for the isolation of RNA. The above mentioned garden soil was autoclaved and 260 g (dry weight) were used for cultivation of plants. After planting of four plants, the beaker glass was covered by a transparent plastic sheet. Inoculation 14 days after planting was performed using a powder duster with 10 mg of uredospores and 10 mg of powdered kaolin for each beaker glass. For mock inoculated variants only 10 mg of powdered kaolin were applied. Plants were inoculated with single spore isolate wxr77 or Kaolin, respectively every hour from 24 to 2 h before leaf sampling to be able to freeze all samples in liquid nitrogen at the same time. Position of beaker glasses were randomized and changed daily to avoid possible effects of location.

### Infection Ratings

All accessions tested for resistance to the different isolates were rated 10 days after the inoculation with leaf rust, when the generation of uredospore pustules on leaves of the susceptible wheat cultivar Thatcher was completed according to McIntosh et al. (1995). This rating system allows the classification as “immune” (rated as “0”), “very resistant” (rated as “;”), “resistant” (rated as “1”), “moderately resistant” (rated as “2”) “moderately resistant to moderately susceptible” (rated as “3”) and “susceptible” (rated as “4”) to leaf rust. The letter “N” has been used to indicate a high degree of necrosis on leaves.

### Staining Procedures and Microscopy

In order to detect the accumulation of H_2_O_2_ in inoculated leaves, DAB was used (Thordal-Christensen et al., 1997). DAB was dissolved in water (1 mg ml^-1^, pH 3.8, HCl) and kept in the dark at 4°C. Leaves of inoculated plants were collected directly before the inoculation (0 hai) and 6, 12, 24, 48, 72, 96, and 168 hai by cutting leaves to segments of 1 cm length. Segments were incubated in reaction tubes (2 ml) in 1.5 ml of the DAB solution for 16 h at room temperature so that the solution was equally dispersed within the entire leaf. Fungal cell walls were stained using Calcofluor White M2R (Rohringer et al., 1977) optimized for the staining of *T. monococcum* leaves. After the DAB stain, leaves were washed twice with deionized water and transferred to reaction tubes (2 ml volume) which contained 1.5 ml of a lactophenol/ ethanol (1:2 v/v) mixture. The lactophenol/ ethanol solution was prepared by mixing 100 g phenol in a solution of 50 ml lactic acid, 100 ml glycerol, 50 ml deionized water and 480 ml of ethanol. Samples were incubated 2 h at room temperature in the lactophenol/ethanol solution and were then boiled for 10 min. The solution was discarded and leaf samples were incubated for 15 min at room temperature in 1.5 ml of a solution of ethanol and water [33.3% ethanol, 66.6% deionized water (v/v)]. The ethanol/ H_2_O solution was discarded and a solution of 0.05 M sodium hydroxide was added. Leaf samples were shaken 15 min, the solution was discarded and samples were incubated 15 min in sterile H_2_O. After the removal of water, 0.1 M Tris-HCl solution was added and samples were incubated for 2 h at room temperature. The solution was discarded and the Calcofluor white M2R solution (0.2% in sterile water, w/v) was added and discarded after 10 min of incubation at room temperature. Samples were washed four times with sterile water, transferred to a microscope slide and embedded in a glycerol/water solution (1:1 v/v). Microscopy of leaf cells and fungal structures was performed using an Axioskop 50, for taking pictures and for the analyses an Axiocam MRc connected with the software package Axiovision 4 (Carl Zeiss AG, Jena) was used. By Calcofluor white M2R, stained fungal structures were observed using the filter set 02 (excitation filter G 365, beam splitter FT 395, and barrier filter LP 420), autofluorescence within plant tissue was recorded using the filter set 05 (excitation filter BP 400-440, beam splitter FT 460, barrier filter LP 470).

For experiments in which fungal structures (hmc, haustoria) were counted, altogether three leaf segments from the middle of the third youngest leaf of three seedlings from three different plants of the same variant were microscopically analyzed. Within each replication 10 infection sites (germinated uredospore and appressorium generated) were counted so that counts of altogether 30 infection sites (3 × 10) were used for the analysis. In order to assess the generation of uredospore pustules in relation to the investigated leaf area, pictures were taken using a stereo microscope (Stemi 2000, Carl Zeiss, Jena, Germany) in combination with the digital camera Axiocam MRc and the software package Axiovision 4 (Carl Zeiss AG, Jena). The DAB stained area has been measured using the magic wand tool within the Adobe Photoshop CS4 Extended version 11.0 in accordance to Li et al. (2007) and Luna et al. (2011) with a tolerance of 110 to determine the entire recorded leaf area and a tolerance of 75 to detect the leaf area which was DAB stained. Measurements were conducted on 10 infection sites per leaf in 3 replications.

### Measurement of H_2_O_2_ Concentration in Leaves

The concentration of H_2_O_2_ was measured in accordance with the methodology described for the eFOX assay by Cheeseman (2006). Samples were collected at the time points directly after the inoculation (0 hai) and 6, 12, 24, 48, 72, 96, and 168 hai. Leaf segments with a size of around 4 cm × 4 cm with a weight of 200 mg were submerged and grinded in liquid nitrogen. The assay mixture contained in accordance to Cheeseman (2006) 250 μM ferrous ammonium sulfate, 100 μM sorbitol, and 100 μM xylenol orange in 25 mM H_2_SO_4_. Following control studies on the influence of various solvents, the assay was modified to include 1% ethanol. In order to avoid changes of the H_2_O_2_ concentration during the experiment 5 mM KCN as an inhibitor of catalase, peroxidase and CuZn-superoxide dismutase was added. Furthermore, to avoid the generation of HCN and the oxidation of H_2_O by free radicals the assay mixture was buffered to a pH of 6.4, a mixture without a leaf sample was used as control and measured after each sample. A standard curve concentration as a function of the wavelength 550 nm was calculated without added H_2_O_2_ and concentrations of 1, 5, 10, 50, 100, 200 and 300 μM H_2_O_2_. This resulted in a nearly linear curve up to a concentration of 200 μM H_2_O_2_. The highest concentration of 300 μM was excluded from the calculation of the concentration curve. In the medium without added H_2_O_2_, the concentration was set to zero. Measurements were replicated three times. For each replication leaf segments from 3 different plants within the inoculated variant at the time points from 0 to 168 hai (see above) were used.

### Protein Extraction and Measurement of Protein Concentration

At the already described time points 200 mg of inoculated and non-inoculated leaves of the resistant accession PI272560 and the susceptible accession 36554 were taken and a protein extraction was performed using a Plant Total Protein Extraction Kit (PE0230, Sigma–Aldrich, Munich, Germany) which is recommended for 10–250 mg of leaf samples, following the instructions, given by the manufacturer.

The amounts and quality of the extracted proteins were tested using the bicinchoninic acid kit for protein determination which works similar to the Lowry procedure (Lowry et al., 1951) and measured in a 96 well plate at an absorbance of 560 nm with a multimode micro plate reader (Sunrise-Basic, Tecan Group, Groeding Austria). A calibration curve (*R*^2^= 0.997, linear range from 0 μg to 4000 μg ml^-1^ protein) using standard solutions from the kit was calculated so that protein concentrations from samples could be determined between 988.3 and 3020.0 μg ml^-1^. If protein concentrations were out of range of the calibration curve, samples were diluted.

### Measurement of Peroxidase Activity

Extracted proteins were diluted to the concentration of the lowest measured protein content of 988.3 μg ml^-1^. 101.8 μl were dissolved in 100 μl of the assay buffer, provided in the peroxidase activity assay kit (MAK092, Sigma–Aldrich), which was used for the determination of the peroxidase activity. Determination was performed following the manufacturer’s instructions; measurements were performed using the plate reader Sunrise-Basic (Tecan Group, Groeding, Austria) at a wavelength of 570 nm. After adding the master reaction mix, the initial measurement was taken 3 min after the incubation in darkness at 37°C in a shaker and then repeated four times every 3 min so that the peroxidase activity could be calculated by the change in measurement from the initial time (Tinitial) to final time after inition (Tfinal) for samples. Firstly the absorption (A) at a wavelength of 570 nm was calculated using the following equation:

$$\begin{array}{c} \Delta A570=(A570)Tfinal-(A570)\mathrm{Tinitial} \end{array}$$

The result was compared with the standard curve to determine the amount of H_2_O_2_ reduced during the assay between Tinitial and Tfinal. The peroxidase activity of a sample could be determined by the following equation at which the amount of H_2_O_2_ reduced between Tinitial and Tfinal (B) is multiplied with the sample dilution factor and then divided by the reaction time (minutes) multiplied with the sample volume (ml, V):

$$\begin{array}{c} \mathrm{Peroxidase}\mathrm{Activity}=\frac{B\times \mathrm{Sample}\mathrm{Dilution}\mathrm{Factor}}{\mathrm{Reaction}\mathrm{Time}\times V} \end{array}$$

The peroxidase activity could be reported as milliunit ml^-1^ where one unit of peroxidase is defined as the amount of enzyme, that reduces 1.0 mmole of H_2_O_2_ per minute at 37°C. The molecular basis for the measurements is the fluorescent peroxidase substrate. The Fe_2_^+^ ion is converted to Fe_3_^+^ ion at acidic pH and the Fe_3_^+^ ion forms a colored adduct with xylenol orange.

### Measurement of Chitinase Activity

The chitinase activity was determined using a chitinase assay kit (CS0980, Sigma–Aldrich). The protein solution (10 μl) with a concentration of 988.3 μg ml^-1^ was measured after 30 min of incubation time with the above mentioned microplate reader at a wavelength of 405 nm. Chitinase activity was calculated using the following equation at which “A405sample” shows the absorbance of the sample at 405 nm, “A405blank” the absorbance of the blank at 405 nm, “0.05” the mmole/ml of *p*-nitrophenol in the standard solution, “0.3” the final volume of the 96 well plate reaction after addition of the stop solution (ml) and “DF” the dilution factor – fold dilution of the original chitinase enzyme or biological solution to prepare sample for the test, “A405standard” showed the absorbance of the standard solution at 405 nm, “time” minutes of incubation and “V_enz_” the volume of the sample (ml):

$$\begin{array}{c} Unitsml^{-1}=\frac{(A405sample-A405blank)\times 0.05\times 0.3\times \mathrm{DF}}{A405\mathrm{standard}\times \mathrm{time}\times \mathrm{Venz}} \end{array}$$

By the manufacturer instructions measurement after 20 min of incubation (a maximum of 30 min) is advised.

### Massive Analyses of cDNA Ends (MACE)

In order to get information on the molecular background of this phr, expression analysis using MACE was performed as described by Zawada et al. (2014) for the identification of proatherogenic pathways in chronic kidney disease. For MACE analyses, RNA was isolated using the NucleoSpin^®^ miRNA kit (Macherey Nagel; Düren, Germany), which allows to separate the large and small fraction of the total RNA. In accordance with the instructions, the large fraction of the total RNA (>200 bp) and the small RNA were isolated and used separately for the preparation of the MACE libraries by GenXpro. RNA was isolated every 2 h from the leaf rust inoculated and mock inoculated leaves up to 24 hai resulting in 48 samples. RNA which was collected between 0 to 8, 8 to 16 and 16 to 24 hai was pooled in one sample after determining RNA concentration so that three samples for the two accessions, each from the inoculated and mock inoculated variant (12 samples), were used for further analyses. The quality of the RNA samples was analyzed running the electrophoretic assay “Plant RNA nano” on a Bioanalyzer (Agilent 2100, Agilent Technologeis, Santa Clara, CA, USA). RNA concentration was measured and the RNA integrity calculated from the electropherogram was between 7.2 and 7.5. The preparation of MACE libraries, the sequencing using an Illumina Hiseq2000 (Illumina Inc, San Diego, CA, USA) with 1 × 100 bps and the quantification of mRNA expression was performed by the GenXpro GmbH (Frankfurt, Germany) according to Zawada et al. (2014).

### Analysis of Resulted Tags

The sequences representing distinct MACE tags were quantified as described by Zawada et al. (2014). Results of the digital gene expression analyses showed the best matching database entry(s) which were identified by annotation to databases like UniProt Knowledgebase (UniProtKB), TIGR Rice Genome Annotation Project (TIGR) and the NCBI Nucleotide database using the Basic Local Alignment Search Tool (Blast) to nucleotides, respectively. All tags (sequences) that could not be annotated to these databases were joined and assembled into “nohit-contigs”. These contigs were annotated to the most suited Swissprot database by BLASTX ^2^. Resulting data consisted of the absolut amount of different tags with matches in sense (S) orientation (5’-3’) and of antisense (AS) tags which matches in AS orientation (3′-5′) to database entries and the description of hits including the matched organism, the amount of homology and the name of the database leading to the hit with best alignments. The total number of tags was used to calculate the frequency of tags, normalized to 1 million tags (tpm) for a simplified comparison of the tags. The *p*-value which defined significant differences between the non-inoculated and inoculated variants of genotypes as well as between the genotypes within the time segments 0–8, 8–16 and 16–24 hai was calculated according to Audic and Claverie (1997). In case no tpm values could be determined to avoid division by zero, the tpm values were set to 0.05. Sequences which were provided by GenXpro were analyzed using Blast2Go (version V2.8.0) working on the basis of a Java web start script (version 1.8.0_25, Oracle Corporation, Redwood Shores, CA, USA) using Blastx procedure with the following settings:

Blast program was set as “BlastX,” the data base (“DB”) was set as non-redundand (nr) so that the best blast hits from 16 databases could be used. The Expect (E-) value was set to < 10^-5^ and the number of Blast hits was set to 20. Xblast resulted in an xml-file and results were analyzed following the descriptions of Conesa and Götz (2008) and Götz et al. (2008) so that GO-terms could be identified. Related to the number of matched GO-terms, differentially expressed genes (determined by the statistical analysis of expression) after an inoculation of the respective accession were identified and aligned to probable proteins and their functions. This procedure was performed for tags which could be detected in the non-inoculated and inoculated variants of PI272560 and 36554. Furthermore, only tags with a factor of fold change >2 or the corresponding log_2_ values, respectively which were significantly different between the non-inoculated and inoculated variants were defined as differentially expressed. From these tags only tags which showed a significantly different tpm value between the accessions in the inoculated variants were used for the identification of GO-terms. GO-terms which explained microscopical observed reactions related to the metabolism of H_2_O_2_, the recognition, response, degeneration of chitin or related to defense responses were used to identify genes in PI272560 and 36554. In order to identify genes which were exclusively expressed in one of the accessions and were possibly involved in resistance, all hits which matched the GO-terms ATP binding (GO:0005524) in combination with the serine/threonine kinase activity (GO:0004674) and/or kinase activity (GO:0016301) and/ or response to chitin (GO:0010200) having a similarity of more than 80% to sequences in databases were taken into account.

### Statistical Analysis of Data

In order to identify differentially expressed genes between the non-inoculated and the inoculated variant, *p*-values describing the probability of a hit to be differentially expressed, were calculated according to Audic and Claverie (1997) using an R-script ^3^. The *p*-value is only provided for pairwise comparisons so that the logarithm to the basis 2 of the ratio of the normalized values of the non-inoculated variants were divided by normalized values of the inoculated variants of the same genotype and time period. Differences between the non-inoculated variant with a *p*-value < 0.05 and an increase or decrease by a factor ≥ 2.0 (logarithm to the basis 2 ≥ 1.0) were used for further comparisons between inoculated and non-inoculated variants. For comparisons between PI272560 and 36554 only tags were used which were identified as significantly expressed between the inoculated and non-inoculated variants of the accessions, and which showed significant different expression levels (*p* < 0.05) in at least one time segment, and hit a GO-term. The haustorial mother cell number, H_2_O_2_ concentation, the peroxidase and endochitinase activity have been compared pairwise on the one hand between the resistant and the susceptible accession within each time point or time segments and on the other hand at different time points within each accession. Due to the fact, that all variants within experiments were replicated three times an ANOVA was performed to detect significant differences between the averages at (α) = 0.05. Statistical calculations were performed using JMP 5.1 Genomics (SAS, Cary, NC, USA).

## Results

### Identification of Highly Virulent *P. triticina* Isolates

As a prerequisite for this study, virulence patterns of six *P. triticina* isolates were determined on Thatcher near isogenic lines (NILs) carrying resistance genes located on the A-genome in order to exclude the involvement of these genes in phr. All isolates investigated were virulent on lines carrying leaf rust resistance genes located on the A-genome, i.e., *Lr10, Lr11, Lr17, Lr20, Lr37* and are partially avirulent against *Lr28* and the resistance gene *Tm* derived from the *T. monococcum* accession Ks92WGRC23. In microscopic analyses on susceptible genotypes (disease score 4), uredospore pustules were observed whereas on the resistant accession PI272560 no uredospore pustules developed independently from the isolate used (**Table 1**). Out of the fungal isolates tested, isolate wxr77 was virulent against all tested *Lr-*genes and produced the highest number of uredospore pustules in accession 36554 (**Table 1**). Hence, this isolate was used for the presented detailed analyses of compatible and incompatible resistance mechanisms in the susceptible accession 36554 and the resistant PI272560, respectively.

**Table 1:** Macroscopic reaction and microscopically visible generation of uredospore pustules of genotypes carrying leaf rust resistance genes (*Lr-*genes) inoculated with different single spore isolates 168 hai.

	Single spore isolates											
Genotype/Thatcher NIL	77wxR		Hk1/3-04		Hk12/3-01		167/176WxR		58WxR		13/20WxR	
Accession/cultivar/NIL	Rating	Pustules mm^-2^	Rating	Pustules mm^-2^	Rating	Pustules mm^-2^	Rating	Pustules mm^-2^	Rating	Pustules mm^-2^	Rating	Pustules mm^-2^
Thatcher	4	5.2 ± 2.5	4	8.0 ± 3.3	4	9.2 ± 2.7	4	7.2 ± 2.0	4	7.8 ± 3.9	4	6.7 ± 0.2
PI272560	0()	0.0 ± 0.0 ^∗^	0	0.0 ± 0.0^∗^	0	0.0 ± 0.0^∗^	0	0.0 ± 0.0^∗^	0	0.0 ± 0.0^∗^	0	0.0 ± 0.0^∗^
36554	3N	4.7 ± 1.1	3N	4.0 ± 1.1^∗^	3N	4.3 ± 0.9^∗^	3N	4.7 ± 1.4^∗^	2N	2.1 ± 1.4^∗^	2N	3.2 ± 1.2^∗^
*Lr10* (Thatcher^∗^6/Exchange)	4	6.7 ± 2.3	4	5.3 ± 2.0	4	5.3 ± 2.9^∗^	4	5.6 ± 3.5	4	6.6 ± 2.1	2-3	3.0 ± 0.3^∗^
*Lr11* (Thatcher^∗^6/Hussar)	2	2.8 ± 0.7^∗^	4	5.6 ± 0.6^∗^	4	5.8 ± 1.3^∗^	4	5.8 ± 1.4^∗^	4	7.7 ± 2.3	4	5.2 ± 0.3^∗^
*Lr17* (Thatcher^∗^6/Klein Lucero)	2-3	2.0 ± 0.9^∗^	4	6.3 ± 3.0	4	6.2 ± 3.0	4	3.9 ± 1.1^∗^	4	5.0 ± 1.9	4	4.5 ± 0.9^∗^
*Lr20* (Thatcher^∗^6/Jimmer)	4	8.6 ± 2.5^∗^	2	2.5 ± 0.4^∗^	4	6.4 ± 3.2	4	5.2 ± 1.6	4	7.6 ± 2.6	4	5.0 ± 2.5
*Lr28* (Thatcher^∗^6/C-77-1)	2	1.2 ± 0.3^∗^	0;	0.0 ± 0.0^∗^	2-3	2.9 ± 0.9^∗^	4	5.6 ± 1.7	4	4.6 ± 1.8	0;	0.3 ± 0.0^∗^
*Lr37* (Thatcher^∗^6/VPM)	4	7.4 ± 1.7	4	4.3 ± 1.5	4	7.1 ± 0.4	4	5.5 ± 0.3	4	7.2 ± 1.8	4	7.3 ± 0.2^∗^
*Tm* (*Triticum monococcum*, Ks92WGRC23)	0;-1	1.0 ± 0.0^∗^	1–2	2.1 ± 0.8^∗^	3	2.4 ± 0.1^∗^	3	3.2 ± 1.2^∗^	1-2	2.3 ± 0.5^∗^	3	3.1 ± 1.5^∗^

*Leaves of genotypes rated without symptoms are displayed by “0,” by “0;” with occasional hypersensitive spots, by “1” with macrosocpic visible hypersensitive spots without uredospore pustules, by “2” leaves with large hypersensitive areas around uredospore pustules, by “3” with uredospore pustules and partial defense reaction on leaves, by “4” with uredospore pustules and no visible necroses or hypersensitive spots around infection sites. “N” was used if large necrotic areas were visible. Asterisks behind the averages of microscopically visible pustules per square mm of 3 replications which consist of three leaves respectively show significant differences of a near isogenic line (NIL) to the susceptible control cultivar Thatcher (α = 0.05).*

### Microscopic Analyses

#### Development of Fungal Structures

**FIGURE 1:**
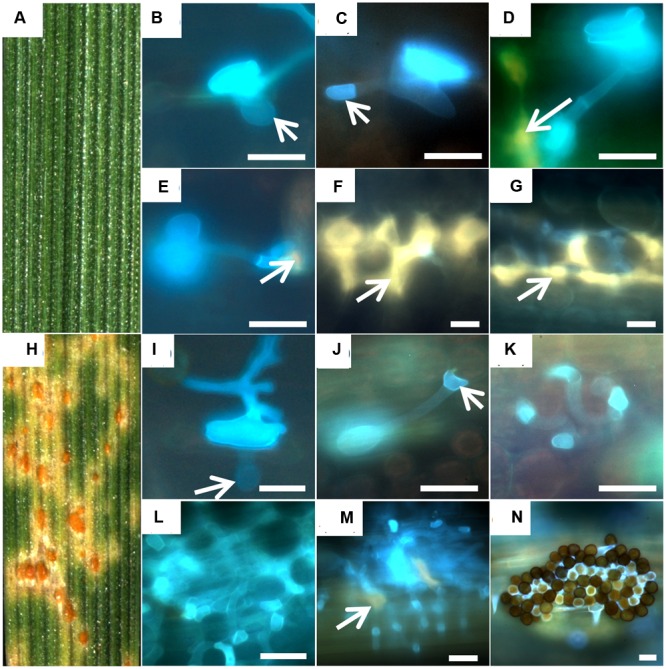
**Development of fungal structures in the resistant accession PI272560** **(A–G)** and the susceptible accession 36554 **(H–N)**. Macroscopic symptoms on leaf segments 168 hai **(A,H)** and microsopic observations 6 hai **(B,I)**, 12 hai **(C,J)**. 24 hai **(D,K)**, 48 hai **(E,L)**, 72 hai **(F,M)**, and 168 hai **(G,N)** are compared. Arrows in **(B,I)** mark the substomatal vesicle, in **(C)** the haustorial mother cell and in **(D,E,F,G,M)** autofluorescence around infection sites. Bars: 20 μm.

**FIGURE 2:**
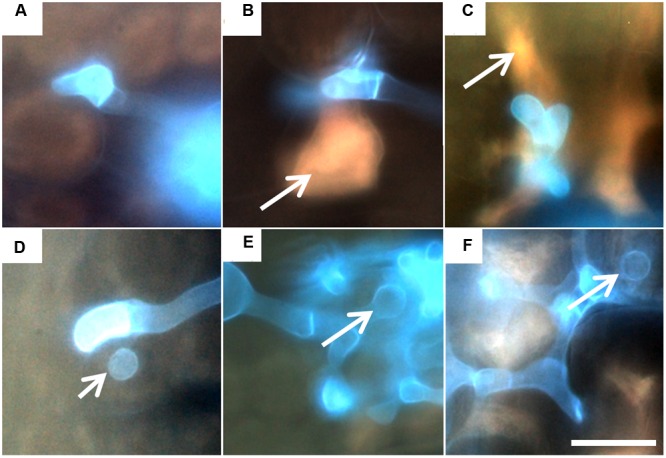
**Generation of haustoria 24, 48, and 72 hai in accession PI272560** **(A,B,C)** and accession 36554 **(D,E,F)**. Arrows in **(B,C)** mark autofluorescence within and around mesophyll cells of PI272560 and haustoria within mesophyll cells of accession 36554 **(D,E,F)**. Bar **(F)**: 10 μm (equal magnification in **A–E**).

While on the resistant accession PI272560 never uredospore pustules developed (**Figures 1A–G**), 4.0 ± 0.6 uredospore pustules per mm^-2^ were observed on leaves of accession 36554 at 168 hai (**Figures 1H,N**; **Table 2**). The microscopic observation of fungal structures revealed the generation of a substomatal vesicle within the substomatal cavity in both accessions 6 hai (**Figure 1**, arrows in B and I). Twelve hai infection hyphae and a haustorial mother cell (hmc) were generated at the cell walls of mesophyll cells (**Figure 1**, arrows in C and J). At 24 hai up to 96 hai the number of hmc around mesophyll cells increased in accession 36554 to 57.1 ± 10.4 but to a much smaller number (4.7 ± 0.6) in PI272560 (compare **Figures 1D–F** with 1K–M; **Table 2**). Only in accession PI272560 autofluorescence around the hmc at 24 hai was observed (**Figure 1D**, arrow), and at all subsequent time points including (48, 72, 96, and 168 hai, **Figure 1**, arrows in D,E,F,G). In the susceptible accession, however, autofluorescence as an indicator of plant defense reactions that could prevent the formation of hmcs was visible to a much lower extent starting at 96 hai (**Figure 1**, arrow in M). In accordance with the lack of autofluorescence, a large number of hmc was formed in accession 36554 at 168 hai, which could not be counted due to the fact that uredospore pustule formation has already begun (**Figure 1N**). The difference between accession PI272560 and 36554 in the number of hmc from 24 to 96 hai was highly significant (**Table 2**). In addition to the development of hmc, the generation of haustoria was also analyzed. Whereas no haustoria were observed in the resistant accession PI272560 (**Figures 2A–C**; **Table 2**), already directly after the formation of the first hmc at 24 hai haustoria were formed in mesophyll cells (**Figure 2D**; **Table 2**) of 36554. The number of haustoria per infection site increased to 5.0 ± 0.2 haustoria per infection site at 72 hai (**Table 2**). In accession 36554 autofluorescence around the infection sites was detected firstly 96 hai, giving hint to a delayed defense reaction which does not prevent the formation of haustoria (**Figures 2B,C,E,F**). Given that not any formation of haustoria in accession PI272560 was observed, it may be concluded that accession PI272560 carries a phr to leaf rust. However, as no autofluorescence was detected earlier than 24 hai in accession PI272560 the mechanism which prevents the formation of hmc cannot be deduced from these microscopic observations.

**Table 2:** Results of the microscopic analyses of the accessions PI272560 and 36554 at 12, 24, 48, 72, 96, and 168 hai with leaf rust.

Hours after inoculation (hai)	Haustorial mother cells		Hmc surrounded mesophyll cells with haustoria per infection site (defined by generated appressorium)	
	PI272560	36554	PI272560	36554
12	0.15 ± 0.05	0.18 ± 0.07	0.0 ± 0.0	0.0 ± 0.0
24	0.58 ± 0.14	0.93 ± 0.16^∗^	0.0 ± 0.0	0.4 ± 0.09^∗^
48	2.9 ± 0.6	6.4 ± 0.8^∗^	0.0 ± 0.0	1.6 ± 0.3^∗^
72	3.5 ± 0.7	25.6 ± 3.9^∗^	0.0 ± 0.0	5.0 ± 0.2^∗^
96	4.7 ± 0.6	57.1 ± 10.4^∗^	0.0 ± 0.0	17.9 ± 6.9^∗^
**Uredospore pustules (mm^-2^)**		
168	0.0 ± 0.0	4.0 ± 0.6^∗^		

*The average of haustorial mother cells and of haustoria was determined on 10 infection sites of three leaves (30 infection sites). The average number of uredospore pustules per mm^2^ from three leaves was determined exclusively at 168 hai. Asterisks mark significant differences of the averages of hmc or haustoria between the accessions (α = 0.05).*

### Hydrogen Peroxide Stain after the Inoculation with Leaf Rust

**FIGURE 3:**
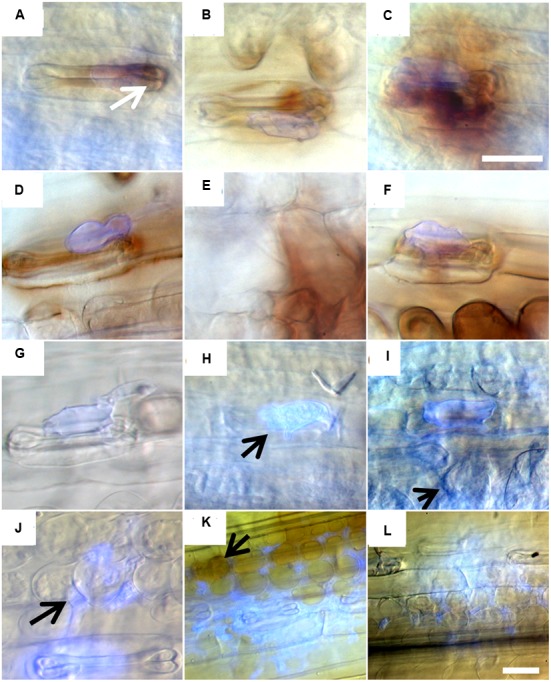
**3,3-Diaminobenzidine stain of hydrogen peroxide around infection sites 6, 12, 24, 48, 72, and 96 hai of accession PI272560 (A–F)** and of accession 36554 **(G–L)**. Arrows show enhanced hydrogen peroxide concentration stained by 3,3 Diaminobenzidine **(A)**, haustorial mother cells (J) and hydrogen peroxide stain by 3,3-Diaminobenzidine around infection of accession 36554 **(K)**. Bar **(C)**: 20 mm (equal magnification in **A–K**), bar **(L)**: 20 μm.

Another mechanism that could prevent the formation of haustoria is the generation of hydrogen peroxide (H_2_O_2_). In order to get first information on the mechanisms involved in this phr, a 3,3 Diaminobenzidine (DAB) stain for hydrogen peroxide (H_2_O_2_) was conducted. The accumulation of hydrogen peroxide was observed 6 hai in infected stomata cells of accession PI272560, already (**Figure 3**, arrow in A) and at all subsequent time points (**Figures 3B–F**). Moreover, in PI272560 the stained area around infection sites was significantly larger than in 36554 at 6, 12, 24, and 48 hai, but a decrease to 537.1 ± 274.8 μm was observed at 96 hai (**Table 3**). In contrast to this, no increase of the DAB stained area was detected in the susceptible accession 36554 during the formation of infection hyphae 6 hai (**Figure 3G**), generation of the substomatal vesicle (12 hai, **Figure 3**, arrow in H) and of hmc formation between 24 to 48 hai (**Figure 3**, arrows in I, J). Increased amount of H_2_O_2_ 72 and 96 hai (**Figures 3K,L**, arrow in K **Table 3**) in 36554 does not result in an effective inhibition of the fungal development. Thus DAB staining indicated that an early increase of the hydrogen peroxide concentration is one of the factors contributing to the phr.

**Table 3:** Average size of 3,3 Diaminobenzidine stained area (μm^2^) around the infection sites at different hai based on 3 replications (10 infection sites per replication).

Hours after inoculation (hai)	DAB stained area (μm^2^) per infection site	
	PI272560	36554
6	1496.2 ± 244.2^∗^	515.9 ± 130.5
12	5321.2 ± 1738.8^∗^	646.3 ± 153.9
24	4253.3 ± 2264.8^∗^	703.5 ± 100.0
48	2455.5 ± 1074.8^∗^	825.2 ± 210.5
72	2304.6 ± 969.6	1,406.0 ± 73.0
96	537.1 ± 274.8	5950.4 ± 1564.6^∗^

*Asterisks show significant differences of stained area between accessions (α = 0.05).*

### Measurement of Hydrogen Peroxide Concentration in Leaves and Peroxidase Assay

**FIGURE 4:**
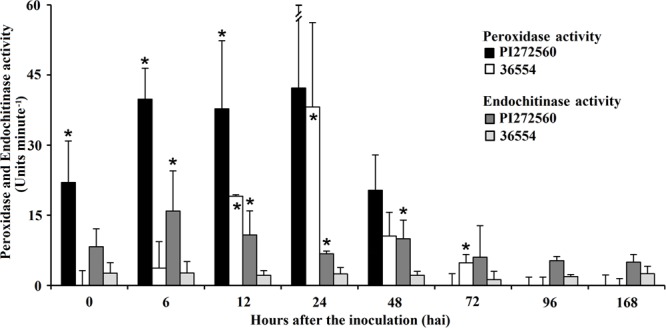
**Peroxidase and Endochitinase activity of accession PI272560 and 36554 directly before the inoculation with leaf rust (0 hai) and 6, 12, 24, 48, 72, 96, and 168 hai.** Units minute^-1^ of peroxidase activity are calculated from the amount (nmole) of H_2_O_2_ reduced at the time point of the final measure (30 min). Units minute^-1^ of endochitinase activity is defined by the time which is necessary to generate 1 μM p-nitrophenol from the appropriate substrate per minute at pH 4.8 at 37°C. Asterisks above columns show significant differences between the accessions, asterisks within columns show differences to the activity at 0 hai after performing ANOVA (α 0.05). Measurements were repeated three times for each accession and time point after inoculation.

Another method to quantify hydrogen peroxide concentrations is the xylenole orange assay. This assay revealed hydrogen peroxide concentrations from 1.60 ± 0.05 (PI272560) and 1.66 ± 0.12 μM (36554) in leaves prior to leaf rust inoculation. The amount significantly increased in leaves of PI272560 12 hai while no significant rise was observed in accession 36554. The highest hydrogen peroxide amount was estimated 48 hai in PI272560 whereas no significant differences were observed in accession 36554 (**Table 4**). The fact that the hydrogen peroxide concentration in PI272560 is significantly higher in comparison to the accession 36554 at 12, 24, and 48 hai, indicates an earlier generation of hydrogen peroxide in the resistant PI272560, which is in accordance with the DAB stained area (**Figure 3**; **Table 3**). The hydrogen peroxide concentration started to decreased at 48 hai in PI272560 to a level at 168 hai which was not significantly different from that observed in accession 36554 and from the non-inoculated control (**Table 4**). Due to the fact that peroxidases are involved in the reduction of hydrogen peroxide, a peroxidase assay on inoculated leaves was performed. Accession PI272560 showed a high peroxidase activity in the non-inoculated control which was at the same level as the activity from 6 to 48 hai and significantly higher than in 36554 up to 12 hai (**Figure 4**). The activity in 36554 increased at 24 hai to the same level as detected in PI272560. After this time in PI272560 the activity decreased suddenly between 48 and 72 hai. Accession 36554, however, showed a consistently lower activity up to 48 hai. The decrease between 48 and 168 hai was delayed in 36554 in comparison to PI272560, so that the activity is significantly higher at 72 hai on a low level of 4.8 ± 1.7 U (**Figure 4**).

**Table 4:** Hydrogen peroxide concentration in μM at different time points after inoculation of three leaves from accessions PI272560 and 36554, respectively.

Hours after inoculation	PI272560	36554
0	1.60 ± 0.05	1.66 ± 0.12
6	1.69 ± 0.12	1.76 ± 0.10
12	1.94 ± 0.05^∗^	1.72 ± 0.07
24	1.93 ± 0.02^∗^	1.75 ± 0.06
48	1.95 ± 0.05^∗^	1.72 ± 0.01
72	1.88 ± 0.20	1.70 ± 0.13
96	1.90 ± 0.34	1.62 ± 0.10
168	1.67 ± 0.07	1.73 ± 0.09

*Significant differences to the non-inoculated control (0 hai) and between the accessions at the same time (α = 0.05) are marked by asterisks.*

### Endochitinase Activity

In microscopy, fungal structures showed reduced fluorescence of chitin containing cell walls 168 hai in accession PI272560 (**Figures 1F,G**) in comparison to 36554 (**Figure 1M**). Therefore, the endochitinase activity (**Figure 4**) was determined in non-inoculated leaves and between 6 and 168 hai. As a first result the activity of endochitinase was not significantly increased in infected leaves in comparison to the non-infected control in accession PI272560, but was in all cases higher than in accession 36554 after inoculation (**Figure 4**). Significantly higher endochitinase activity was detected in PI272560 at 6, 12, 24, 48, and 96 hai in comparison to accession 36554. The peak of activity 6 hai suggests that an early enhanced endochitinase activity is involved in the effective inhibition of fungal development in accession PI272560 which is in accordance with results obtained by DAB staining revealing an increased concentration of hydrogen peroxide at 6 hai in accession PI272560 (compare **Figure 3A** with **3G**).

### Results of the Massive Analysis of cDNA Ends (MACE)

#### Assignment and Determination of Tags

The higher concentration of hydrogen peroxide (**Table 4**, **Figure 3**), of phenolic compounds (**Figure 1** arrows in D, F, G and **Figures 2B,C**) and the increase in peroxidase and endochitinase activity already 6 hai (**Figure 4**) suggests that an early defense reaction occurs before autofluorescence surrounding the infection sites is microscopically visible. To investigate the molecular mechanisms underlying this resistance reaction, MACE transcription profiles were produced from leaves of the inoculated and non-inoculated variants of the susceptible and resistant accession sampled at three time segments 24 hai (0–8, 8–16, and 16–24 hai). In summary 515028 MACE tags (PI272560) and 570787 (36554) were successfully assigned by homology to entries in public data bases. In order to identify genes or related proteins which are involved in the phr of PI272560 or the delayed or missing defense reaction in 36554, investigations focused on transcripts/genes that were differentially expressed between the non-inoculated and inoculated variants, respectively, at the three time intervals. As shown in Supplementary Table S1, between 3462 and 7128 tags were determined as genes differentially expressed between the non-inoculated and inoculated variants respectively in the two accessions.

### Association of Differentially Expressed Tags to Gene Ontology Terms

From differentially expressed genes between 603 (non-inoculated) and 1430 (inoculated) sequences were associated with GO-terms, using the BlastX procedure (Supplementary Table S1). Of these, 463 were differentially expressed between the non-inoculated and inoculated variants in both, PI272560 and 36554. From these sequences 311 turned out to be significantly (*p*-value < 0.05) differentially expressed between the accessions. At the first time segment from 0 and 8 hai, 42 sequences which matched GO-terms and were identified in PI272560 and 36554 showed a differential expression between respective accessions whereas 8–16 hai 151 sequences and 16–24 hai 118 sequences turned out to be differentially expressed (Supplementary Table S2). These sequences (shown in Supplementary Table S3) were annotated and associated via BlastX to 345 different GO categories (specified in Supplementary Table S4). The most commonly matched GO-terms in each of the time segments comprise the functions “metal ion binding” (GO:0046872, matched 63 times), and “membrane” (GO:0016020, matched 58 times).

### Peroxidases and Catalases

In accordance with the observed differences of the hydrogen peroxide concentration within the 3 investigated time segments and the different peroxidase activity on the basis of the GO-terms “oxydation-reduction process” (GO:0055114, matched 57 times), “oxidoreductase-activity” (GO:0016491, matched 56 times, Supplementary Table S5) in combination with the GO-term “response to oxidative stress” (GO:0006979) 15 differentially expressed sequences corresponded to 6 different peroxidases, one catalase and two uncharacterized or predicted proteins. All peroxidases (*Peroxidase 6, Peroxidase 1, Peroxidase 54* and a *Peroxidase* precursor) were expressed at all time points (**Supplementary Figure S1**) at least in PI272560. However, they were generally differentially expressed between the accessions at different time segments and were mostly up-regulated or exclusively expressed in PI272560 like the *Peroxidase* precursor (2.77 tpm). Generally, in response to infection peroxidases were first up-regulated in PI272560. For example, in PI272560 *Peroxidase 6* was up-regulated already from 0 to 8 hai (**Supplementary Figure S1**) whereas no peroxidase was up-regulated in 36554 at this early time point. Furthermore, *Peroxidase 6* showed the highest difference between the two accessions with the exception of an uncharacterized protein within this time segment (Supplementary Table S3). At the time segment from 8 to16 hai out of four identified peroxidases, *Peroxidase 6* was the only one showing a significantly higher expression in PI272560. As an indicator for a delayed peroxidase expression in 36554 in comparison to PI272560 all other identified peroxidases in particular the class III peroxidase *Prx113* and *Peroxidase 1* showed a higher tpm value in accession 36554 (Supplementary Table S3) from 8–16 hai to 16–24 hai. The higher tpm values of peroxidases within the earliest time segment indicate a fast defense response within the first 8 hai in the resistant accession PI272560.

### Chitinases

The already mentioned enhanced chitinase activity in PI272560 indicated the involvement of genes within the GO-category “chitinase activity” (GO:0004568). This GO-term matched 8 genes, comprising a predicted protein and 3 chitinases from which two were homologous to a *Chitinase 2* of wheat. Another matched to a chitinase of *Hordeum vulgare* (Supplementary Table S2). These chitinases were identified within all time segments and showed significantly enhanced tpm values in PI272560 with the exception of the time segment from 8 to 16 hai in which no differences were observed for the chitinase from *H. vulgare* (comp1098, **Supplementary Figure S2**). In accordance with the chitinase enzyme assay, the number of tags was highly significantly enhanced within the first time segment in PI272560 (269.7 tpm, 36554: 14.8 tpm, Table S3) for the *H. vulgare* chitinase (comp1098) and also for one of the *Chitinase 2* genes (comp13351) with 4.86 tpm in PI272560 (36554: 0.49 tpm). The comparison between the non-inoculated and inoculated variants of PI272560 and 36554 showed on the one hand a significantly increased (or lower decreased) expression of chitinases in PI272560 in which on the other hand an enhanced tpm value within all time segments was observed (**Supplementary Figure S2**). However, the decreased expression in both accessions 0–8 hai does not explain the highly different tpm values within this time segment between PI272560 and 36554, i.e., for the above mentioned *H. vulgare* chitinase (comp1098, **Supplementary Figure S2**, Supplementary Table S3) so that an early or constitutive enhanced expression rate of chitinases in the resistant accession is indicated (**Supplementary Figure S2**). This again supports the concept of rapid induction of defense reactions in the resistant accession PI272560.

### Glucanases

Interestingly all chitinases hit in addition the GO-term “carbohydrate metabolic process” (GO:0005975) which is also matched by all eight differentially expressed (endo)-β-1,3-glucanases from which 3 showed a similarity lower than 70% to glucanases in databases (Supplementary Table S3). A sequence matching a β-Glucanase from *H. vulgare* with a similarity of 90% (TC452276) was identified to be differentially expressed within 8–16 and 16–24 hai and showed an enhanced tpm value in the inoculated variants of the susceptible accession (**Supplementary Figure S3**). These glucanases were detected in all time segments and similar to the peroxidases and chitinases the tpm values were mostly higher shortly after the inoculation in PI272560 but are down regulated at later time points (**Supplementary Figure S3**).

### Additional Genes Involved in Defense Reactions

Surprisingly the GO-terms “defense response”(GO:0006952) “defense response to fungus” (GO:0050832) and “plant-type hypersensitive response” (HR; GO:0009626) are matched only by 13 sequences from which one was a hypothetical and uncharacterized protein (comp18958), another related to a seven transmembrane protein with similarity to *Mlo6* from *T. aestivum* (TC274970) and *Mlo2* (TC397596). Both were upregulated at all time segments in accession 36554 (Supplementary Table S2, **Supplementary Figure S4**). In contrast to this, all hits related to *Mlo* are down regulated at all time segments in accession PI272560. *T. aestivum Pr4* and *H. vulgare Pr10* showed an enhanced tpm value in 36554 in the late time segment, while an increased expression in the early time segments (0–16 hai) was observed in PI272560e segment. Surprisingly, the Hypersensitive-induced reaction gene 3 (*HIR3*) showing a higher expression in PI272560 does not match to the GO-term “HR” (Supplementary Table S2, **Supplementary Figure S4**). All genes identified as differentially expressed between the inoculated variants, i.e., peroxidases, chitinases, glucanases, *Pr*-genes, and the *HIR3* gene showed a higher tpm amount in the earliest time segment in PI272560 than in 36554. *Pr10* and the *HIR3* gene turned out to be expressed to a higher level from 16 to 24 hai in accession 36554 (**Supplementary Figure S4**). Other *Pr*-genes, matching the GO-terms “defense response” (GO:0006952) and “defense response to fungus” (GO:0050832) were completely similar to the *Pr1* family (Supplementary Table S2). Sequences similar to these genes showed inconsistent and mostly non-significantly different expression levels. In accession 36554 the *Pr1* precursor expression increased significantly 16–24 hai in inoculated variants in comparison to the non-inoculated control and Pi272560, but PI272560 revealed higher tpm amounts than 36554 16–24 hai. Like other defense relevant genes all hits related to the *Pr1* family showed significantly enhanced tpm amounts 8 hai in PI272560 (**Supplementary Figure S4**). After the analysis of all hits to GO-terms which are related to reactions observed microscopically and in additional assays (see Figures) it may be concluded, that the delayed expression of the genes mentioned above in 36554 results in susceptibility and that the lower defense reaction at later time points observed for some genes does not prevent the generation of uredospore pustules. On the other hand, the early and high expression of the observed genes in accession PI272560 within the first 8 hai (Supplementary Table S2) leads to the complete inhibition of the haustoria generation so that a phr is present in PI272560.

### Genes Exclusively Expressed in One of the Genotypes

Genes which are not differentially expressed between the inoculated and inoculated variants but showed constitutive expression exclusively in the respective accession and may be therefore also involved in defense reactions have been detected by the identification of hits which included the GO-term “ATP binding” (GO:0005524) in combination with the “serine/threonine kinase activity” (GO:0004674) and/or “kinase activity” (GO:0016301) and/or “response to chitin” (GO:0010200). Using these GO-terms, 3 proteins could be detected in PI272560 and 4 in 36554 (Supplementary Table S5). The sequences detected in PI272560 showed highest homology to a chloroplastic Phosphoribulokinase from *Beta vulgaris*, a Calcium-dependent protein kinase 28 (*Aegilops tauschii*) and a serine/threonine protein kinase which has been identified as a Putative cysteine-rich receptor-like protein kinase 20 from *Triticum urartu.* In accession 36554 Receptor-like protein kinase *HSL1* (*Aegilops tauschii*), Lectin receptor kinase (*Triticum aestivum*), Calcium-dependent protein kinase 3 (*Aegilops tauschii*) and a predicted protein (*Hordeum vulgare* subsp. *vulgare*) with similarities to a Serine/threonine-protein kinase *SAPK10* from *O. sativa* were identified. The Phosphoribulokinase detected in PI272560 matched in addition to the GO-term “response to chitin” and other GO-terms related to resistance against fungal pathogens: “defense response to fungus” (GO:0050832), “regulation of hydrogen peroxide metabolic” process (GO:0010310) and surprisingly “systemic acquired resistance” and “salicylic acid mediated signaling pathway” (GO:0009862). All other hits in 36554 were most frequently related to “stomatal movement” (GO:0010118, GO:0010119) and “response to salt stress” (GO:0009651). The fact that only one of these hits could be dedicated to the GO-term “defense response to fungus” suggest that the detected Phosphoribulokinase protein is involved in the early phase of the resistance reaction in PI272560 whereas other matched GO-terms which are related to abiotic stress could be a response to the fungal infection which affects stomatal and mesophyll cells within the first 24 hai (Supplementary Table S5). These genes showed low tpm amounts between 0.16 ± 0.09 and 1.70 ± 0.74 without any significant differences between non-inoculated and inoculated variants or time segments of respective accessions.

## Discussion

**FIGURE 5:**
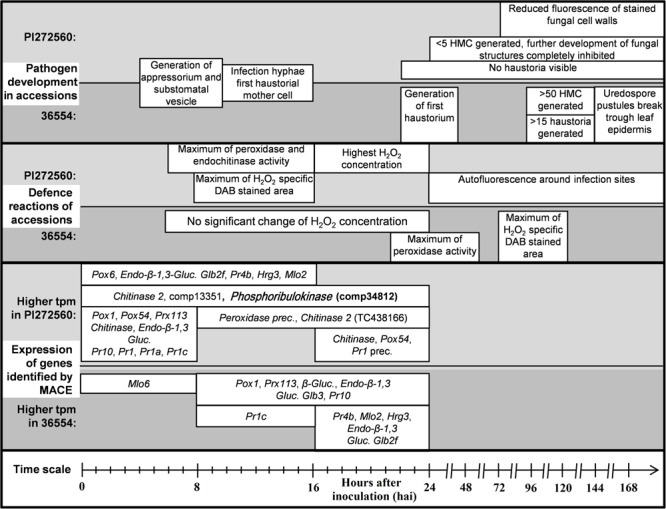
**Overview of the fungal development and determined defense reactions of the accessions PI272560 and 36554.** Fungal structures, i.e., haustorial mother cells are abbreviated with “HMC” and Diaminobenzidine with “DAB.” Differentially expressed genes are displayed within the time segments of higher expression of the respective accession. Peroxidases are abbreviated with “Pox,” class III peroxidases with “Prx,” Glucanases with “Gluc.,” pathogenesis related genes with “Pr,” the Hypersensitive reaction gene with “HIR,” Mildew resistance locus o with “Mlo” and precursors of genes with “prec.”. Exclusively in one of the accessions expressed genes which could be identified as in defense reaction of plants involved are printed in bold letters.

In the present work the resistance reaction of *Triticum monoccocum* accession PI272560 to leaf rust in comparison to a susceptible *T. boeoticum* accession on the phenotypic, physiological and molecular level was characterized. This comprehensive analysis led to the conclusion that rust resistance in PI272560 is a pre-haustorial resistance characterized by a rapid HR resulting in the non-formation of hmcs which are essential for effective infection. Resistance is directed against all tested races of *P. triticina* which in no case caused any microscopically visible uredospore pustule development. Thus, PI272560 may be a promising source of resistance that may replace the currently widely used monogenic, *Lr*-gene-based, race-specific resistances against *P. triticina* in wheat that are always in danger to be overcome by emerging new races. Today, more than 70 resistance genes (*Lr*-genes) against rust in wheat are known (Imbaby et al., 2014, reviewed by McCallum et al., 2016) but only a few *Lr*-genes were transferred to wheat cultivars as single genes or in combination (McIntosh et al., 1995; McCallum et al., 2016). However, most of these *Lr*-genes are race-specific in the sense of the gene-for-gene hypothesis (Flor, 1956). The use of these monogenic resistances in widely grown cultivars selects for pathogen races with complex virulence patterns (Kolmer et al., 1991, 1995). This was recently demonstrated by the breakdown of *Lr37*-based race specific resistance in wheat cultivars grown on about 45% of the European wheat acreage (Goyeau and Lannou, 2011). Similarly, *Lr26* based resistance was overcome by the isolate SaBa77 (Bartoŝ et al., 1996; Serfling et al., 2011). A pressing demand therefore exists for durable, non-race specific leaf rust resistances. Monogenic, race specific *Lr*-genes like Lr1 (Cloutier et al., 2007), *Lr10* (Feuillet et al., 2003), *Lr19* (Gennaro et al., 2009), and *Lr21* (Huang et al., 2003) code for classical CC-nucleotide-binding site–leucine-rich repeat (CC–NBS–LRR) receptor-like protein kinases (RLPK) (Sela et al., 2012). Such proteins induce a HR including activation of calcium and ion fluxes, an oxidative burst, mitogen-associated protein kinase cascades and induction of pathogenesis-related genes (reviewed by McHale et al., 2006; Eitas and Dangl, 2010; Bernoux et al., 2011). These defense reactions occur after haustoria are formed (Southerton and Deverall, 1989). In contrast, *Lr34* which is mainly used in cultivars in combination with other *Lr*-genes (McCallum et al., 2016) codes for an ATP binding cassette transporter. Similar to *Lr46* and *Lr67* it confers non-race-specific, quantitative resistance characterized by slow rusting (Krattinger et al., 2009, 2011; Risk et al., 2012). But, none of these resistances leads to a prehaustorial abort of the fungus as was shown for accession PI272560 in our study (Lagudah, 2011; Herrera-Foessel et al., 2014; Lin et al., 2014). Resistance to *Puccinia graminis and P. triticina in Triticum monoccocum* was firstly described by The (1973). Niks and Dekens (1991) then observed prehaustorial and posthaustorial resistance in *T. monococcum, T. boeoticum and T. urartu*. phr is described as a non-host resistance without HR and has been observed, e.g., in the pathosystems *T. monococcum* and *P. triticina* or T. aestivum and rye-specific isolates of *P. triticina* (Niks, 1983). However, the microscopic analyses of accession PI272560 showed clearly that the generation of haustoria was completely inhibited whereas in the susceptible *T. boeoticum* accession haustoria were generated. In *T. boeoticum* also weak signs of a HR like autofluorescence 72 hai were observed (compare **Figures 2A–F**, summarized in **Figure 5**) so that the classification as a near non-host according to the definition by Bettgenhaeuser et al. (2014) cannot completely be excluded. However, accession 36554 was the most susceptible line of investigated diploid wheat relatives, which were all susceptible to *P. triticina* (data not shown). This is in accordance with Anker and Niks (2001) who stated that *T. boeoticum* accessions are susceptible to *P. triticina*. However, accessions showed weak posthaustorial resistance reactions with different effects on the infection process. This is confirmed by the macroscopic data (**Table 1**), because accession 36554 turned out to be susceptible to the isolate wxr77 but showed microscopically weak defense reactions (**Table 1**; **Figure 1M**). In previous studies (Niks and Dekens, 1991; Anker and Niks, 2001) samples were only analyzed at 42 hai. However, a HR in mesophyll cells which were in contact with fungal haustorial mother cells in accession PI272560 was observed already at 24 hai(compare **Figure 1D** with 1K). Hence it can be concluded that phr at least in the resistant accession PI272560 is due to an early HR of the first infected mesophyll cells (**Figure 2B**, autofluorescence) that may have been missed in the previous studies. A HR accompanied by H_2_O_2_ accumulation also occurs in other interactions of plants with fungal parasites, as e.g., in the interaction of barley with powdery mildew (Thordal-Christensen et al., 1997) and causes non-host resistance to wheat stripe rust in broad bean (Cheng et al., 2012). The efficiency of non-host resistance in accession PI272560 is confirmed by the phenotype which was completely green without any lesion or macroscopically visible hypersensitive spots (compare **Figures 1A,H**). Furthermore, the resistance of this accession to all investigated leaf rust isolates carrying different virulence patterns supports the assumption that it resembles non-host resistance. This type of resistance prevents potentially phytopathogenic microorganisms to infect any cultivar of an incompatible plant species (reviewed by Nuernberger and Lipka, 2005; Bettgenhaeuser et al., 2014). Besides the macroscopic and microscopic evaluation, transcription profiling revealed which genes were responsible for the physiological changes that were observed and also presumably explained parts of the vulnerability of the susceptible accession. Non-host resistance reactions had been investigated before on the molecular level by qPCR by Cheng et al. (2012). However, they focused their analysis on a few pathogenesis related genes like peroxidases, β-1,3 glucanases and chitinases. The MACE technology, however, that was applied for the first time for the analysis of the transcriptome responses of plants to an infection by fungal pathogens delivers a complete overview of the regulation of almost all genes active in the infected leaves. MACE has been successfully applied in medical research (Müller et al., 2014; Zawada et al., 2014; Nold-Petry et al., 2015) and for the analysis of the translation and stability of messenger RNA (Müller et al., 2014). As we demonstrate here, it is also well suited to characterize defense mechanisms and hint to candidate resistance genes in plants. In our studies, peroxidases, β-1,3 glucanases, chitinases and Pr-genes were up-regulated very early in the resistant acesssion in the *T. monococcum* – *P. triticina* interaction within the first 24 hai and in particular within up to 8 hai (summarized in **Figure 5**). Similar genes were up-regulated in the susceptible accession, however, much later, when hmc were already generated around the mesophyll cells. Genes which were most up-regulated after the pathogen attack, like the observed peroxidases have been also described in the context of non-host resistance, e.g., for barley- *B. graminis* f. sp. *tritici* and *P. triticina* (González et al., 2010). Also, the increased expression of chitinases at all investigated time segments in PI272560 underpins the results of endochitinase measurements that indicated a significant increase in activity of the enzyme (**Figure 5**). A correlation of high chitinase expression with resistance has been described by others for wheat genotypes showing resistance against leaf rust (Van Loon and Van Strien, 1999; Anguelova-Merhar et al., 2001; Mohammadi et al., 2001). Also, chitinase over-expressing rice transgenics were resistant to powdery mildew (reviewed by Srivastava and Raghav, 2014). However, chitinases confer resistance only in combination with other defense related genes (reviewed by Grover, 2012) so that a combination of differentially expressed pathogenesis-related genes must be the reason for the resistance of PI272560. Interestingly in particular *Pr4* related proteins have chitinase activity inhibiting the growth of fungal hyphae (Caruso et al., 1999; Bertini et al., 2003). Hence, also with respect to the results of the endochitinase enzyme assay reduced fluorescence of chitin as the main component of the fungal cell wall detected in the resistant accession may be explained by the enhanced chitinase activity in PI272560. Additional genes like β-1,3-glucanases are also known to be involved in non-host reactions (Ham et al., 1991) and showed a higher expression soon after the infection in other pathosystems (Shetty et al., 2009). These expression data explain microscopic observations which showed that indeed very few haustorial mother cells are generated 12 hai in Pi27560 but their growth is inhibited in contrast to the susceptible accession. Subsequently penetration hyphae were aborted, before haustoria were established in the mesophyll cells (**Figure 2**; **Table 2**). Beside these well documented defense genes that were rapidly up-regulated in the resistant accession, two 7 transmembrane proteins with homology to *Mlo2* and *Mlo6* protein genes from wheat (Konishi et al., 2010) were up-regulated in the susceptible accession (**Supplementary Figure S4**). Mlo proteins are negative regulators of plant defense reactions as, e.g., cell death and other responses to biotic and abiotic stresses in epidermal cells and thus mediate the plant’s susceptibility to pathogens (Piffanelli et al., 2002; Yu et al., 2005; Várallyay et al., 2012). Also in the susceptible accession up-regulation of the Mlo homolog might be one reason for its delayed defense reaction and could explain the continuous development of fungal structures visible in the microscope. In the resistant accession however, the expression of the transcript for a “Hypersensitive induced response protein (HIR) 3” which was implicated in the inhibition of stripe rust proliferation during an incompatible interaction with wheat plants (Yu et al., 2008; Qi et al., 2011) increased. HIRs alter the expression levels of defense-related genes and lead to the accumulation of ROS, elicit HR, and enhance defense responses against biotrophic pathogens like rust (Bozkurt et al., 2010; Duan et al., 2013). Furthermore, *Pr10* may be involved in phr. *Pr10* is involved in the incompatible interaction between wheat and stripe rust (Zhang et al., 2010), and in the non-host resistance of broad bean to wheat stripe rust (Cheng et al., 2012). In this study *Pr10* was up-regulated as early as 12 hai, so that the reaction of PI272560 is in accordance with non-host resistance described by Cheng et al. (2012). Transcripts related to the *Pr1* family were identified also in other plant species like barley (Van Loon and Van Strien, 1999) or grapevine (Li et al., 2011). Rauscher et al. (1999) postulated the inhibition of fungal differentiation as a possible effect of *Pr1* proteins, and presented evidence, that apices of infection structures might represent the target of these anti-fungal proteins so that a higher concentration at the time when hyphae come in contact with mesophyll cells can lead to the early arrest of the infection in PI272560. The initiation of non-host resistance elicited by pathogen/microbe associated molecular patterns (PAMPs/MAMPs) of *P. triticina*, which are recognized at the stage of infection hyphae development prior to penetration of cells, is also known in other non-host rust interactions. Jafary et al. (2008) concluded that the immunity of barley (Hordeum vulgare) against *P. triticina* is due to the perception of PAMPs in case the basal immune response is not suppressed by effectors. Furthermore, peroxidases have been identified as involved in basal resistance in this pathosystem (Jafary et al., 2008). In other non-host rust interaction, e.g., broad bean to wheat stripe rust (Cheng et al., 2012), Brachypodium distachyon–Puccinia emaculata (rust of switchgrass, Gill et al., 2015) or Arabidopsis thaliana and wheat stripe rust (Cheng et al., 2013) increased expression of Pr-genes including chitinases and glucanases were detected. In conclusion defense reactions in other non-host rust interactions are comparable to our microscopic observations, H_2_O_2_ measurements and peroxidase and chitinase assay results and are well explained by the MACE transcription profiles that revealed an increased expression of defense related peroxidase, chitinase and β-1,3 glucanase genes at very early time points after infection. Moreover, MACE results rule out the expression of *Lr*-genes in either accession and thus exclude the contribution of this already known resistance pathway to resistance of accession PI272560. Since *Lr*-genes are excluded as possible resistance-conferring genes other candidate genes must be responsible for the observed, efficient non-host resistance in PI272560. Such candidate genes should be up-regulated or exclusively expressed in PI272560 already at very early time points after infection, and may be categorized in GO-categories related to resistance against pathogens. One of these is a Phosphoribulokinase (Supplementary Table S5) similar to the one identified by Zhao et al. (2014) as related to a non-host reaction of rice to stripe rust. However, it is very unlikely that an individual gene is responsible for the observed non-host resistance. However, for none of the other genes exclusively expressed in Pi27256 and listed in Supplementary Table S5, evidence for their involvement in resistance to obligate biotrophic fungal pathogens and to leaf rust in particular exists. Genes that were up-regulated in accession 36554 non-specifically participate in biotic stress tolerance also in other pathosystems (Kobayashi et al., 2004; Bouwmeester and Govers, 2009). If strongly up-regulated they may induce HR and protein kinase activation (Romeis et al., 2001). However, this was not observed in accession 36554. Non-host resistance is usually accompanied by early H_2_O_2_ production and metabolism as, e.g., in the pathosystem O. sativa - wheat stripe rust (Zhao et al., 2014). In cowpea infection with non-pathogenic rust fungi led to arrest of infection hyphae immediately after the formation of haustorial mother cells and autofluorescence, deposition of phloroglucinol/HCl-positive substances in plant cells contacting them as well as phenylalanine ammonia-lyase and extracellular peroxidase activity as early as 10 and 24 hai was detected (Fink et al., 1991). Also in the A. thaliana-coffee leaf rust (Hemileia vastatrix) non-host pathosystem fungal growth was arrested at an early stage of stomatal infection, the expression of Pr-, and peroxidase genes and several other defense-related genes was increased, and phenolic substances and callose accumulated at the infection site (Azinheira et al., 2010). Data from barley suggest that non-host resistance to heterologous rust species including *P. triticina* is controlled by QTL with different and overlapping specificities and by an occasional contribution of an R-gene for hypersensitivity (Jafary et al., 2008). Also, Niks and Marcel (2009) demonstrated that in each population from a cross between non-host species that show natural variation with respect to the degree of resistance to a non-host pathogen, a different set of genes explains the resistance to heterologous rusts. They concluded that non-host plant genotypes may contain redundant defense-related genes so that the neutralization of only one of them should not result in a selective advantage for the mutant pathogen. These observations in other pathosystems resemble our results from the PI272560-rust pathosystem and support our assumption that the resistance of PI272560 to rust is a non-host resistance. It can be concluded that the phr of accession PI272560 is non-race specific and efficient against all *P. triticina* isolates analyzed. The rapid onset and the complexity of the defense response suggest the involvement of additional resistance genes that need to be identified in future work. In this respect the MACE transcription profiling technology that was applied here for the first time for the elucidation of plant reactions to fungal pathogens should be helpful as it is precise and enables the quantification of transcripts. The efficiency of the described non-race specific resistance renders it a formidable candidate for broad application in wheat breeding programs for durable leaf rust resistance in the future.

## Author Contributions

AS and FO designed the experiments. AS and PW conducted the experiments and analyzed the data. ST contributed to statistical analyses. AS, PW, and FO wrote the manuscript. All authors agree to be accountable for all aspects of the work in ensuring that questions related to the accuracy or integrity of any part of the work are appropriately investigated and resolved. All authors contributed to and approved the final manuscript.

## Conflict of Interest Statement

The authors declare that the research was conducted in the absence of any commercial or financial relationships that could be construed as a potential conflict of interest.

## Footnotes

1 http://www.usask.ca/agriculture/plantsci/winter_cereals/winter-wheat-production-manual/chapter-8.php

2 www.pelobiotech.com/files/vortrag_genxpro_in_h_14_08_12.pdf

3 http://search.cpan.org/∼scottzed/Bio-SAGE-Comparison-1.00/lib/Bio/SAGE/Comparison.pm

## Supplementary Material

FIGURE S1 **Expression of genes matching the GO-terms “oxydation-reduction process” (GO:0055114), “oxidoreductase-activity” (GO:0016491) and “response to oxidative stress” (GO:0006979) which were identified in at least one time segment as significantly differentially expressed between the inoculated accessions PI272560 and 36554 on the basis of tags per million (tpm, *p* < 0.05) and which were differentially expressed between the non-inoculated and inoculated variant of at least one of the accessions (*p* < 0.05, log_2_ fold change > 1).** Specifications outside the columns show the *p*-value of the expression differences between the inoculated and the non-inoculated variant of the particular accession. Asterisks and specifications above columns show significant higher values of tags per million (tpm) in the inoculated accession PI272560, asterisks below columns in the inoculated accession 36554. Non-significant differences are abbreviated with “n.s.”

FIGURE S2 **Expression of genes matching the GO-term defining “chitinase activity” (GO:0004568) which were identified in at least one time segment as significantly differentially expressed between the inoculated accessions PI272560 and 36554 on the basis of tags per million (tpm, *p* < 0.05) and which were differentially expressed between the non-inoculated and inoculated variant of at least one of the accessions (*p* < 0.05, log_2_ fold change > 1).** Specifications outside the columns show the *p*-value of the expression differences between the inoculated and the non-inoculated variant of the particular accession. Asterisks and specifications above columns show significant higher values of tags per million (tpm) in the inoculated accession PI272560, asterisks below columns in the inoculated accession 36554. Non-significant differences are abbreviated with “n.s.”.

FIGURE S3 **Expression of genes matching the GO-term “carbohydrate metabolic process” (GO:0005975) which were identified in at least one time segment as significantly differentially expressed between the inoculated accessions PI272560 and 36554 on the basis of tags per million (tpm, *p* < 0.05) and which were differentially expressed between the non-inoculated and inoculated variant of at least one of the accessions (*p* < 0.05, log_2_ fold change > 1).** Specifications outside the columns show the *p*-value of the expression differences between the inoculated and the non-inoculated variant of the particular accession. Asterisks and specifications above columns show significant higher values of tpm in the inoculated accession PI272560, asterisks below columns in the inoculated accession 36554. Non-significant differences are abbreviated with “n.s.”.

FIGURE S4 **Expression of genes matching the GO-terms “defense response” (GO:0006952) “defense response to fungus” (GO:0050832) and “plant-type hypersensitive response” (GO:0009626) which were identified in at least one time segment as significantly differentially expressed between the inoculated accessions PI272560 and 36554 on the basis of tags per million (tpm, *p* < 0.05) and which were differentially expressed between the non-inoculated and inoculated variant of at least one of the accessions (*p* < 0.05, log_2_ fold change > 1).** Specifications outside the columns show the *p*-value of the expression differences between the inoculated and the non-inoculated variant of the particular accession. Asterisks and specifications above columns show significant higher values of tpm in the inoculated accession PI272560, asterisks below columns in the inoculated accession 36554. Non-significant differences are abbreviated with “n.s.”, the Hypersensitive-induced reaction protein 3 with “HIR3.”
