# Supplementary figures and images for: Microscopic and Molecular Characterization of the Prehaustorial Resistance against Wheat Leaf Rust (Puccinia triticina) in Einkorn (Triticum monococcum)
Source: Front Plant Sci. 2016 Nov 9;7:1668. doi: 10.3389/fpls.2016.01668 (PMC5101855; doi:10.3389/fpls.2016.01668)

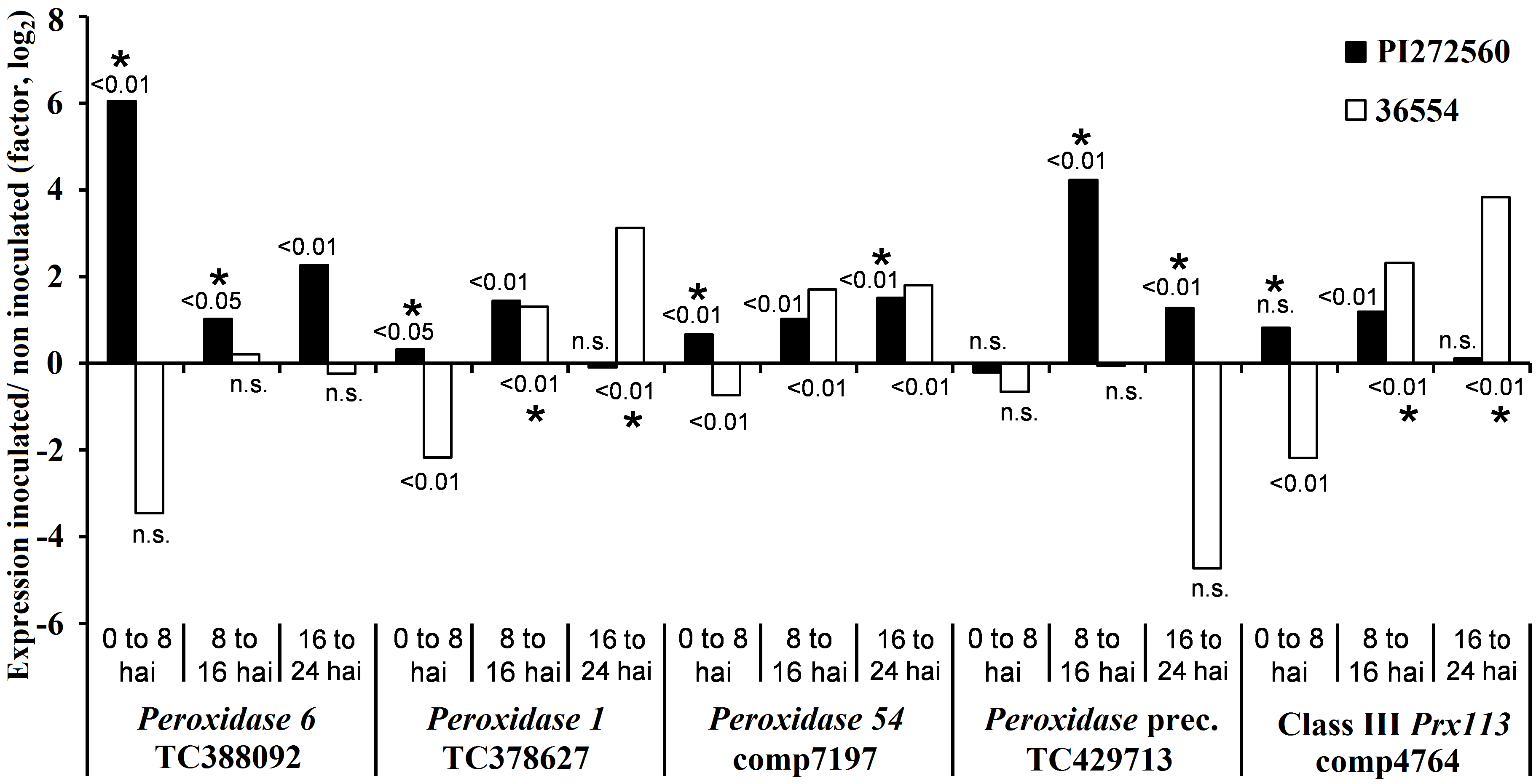

Supplement: FIGURE S1 — Expression of genes matching the GO-terms “oxydation-reduction process” (GO:0055114), “oxidoreductase-activity” (GO:0016491) and “response to oxidative stress” (GO:0006979) which were identified in at least one time segment as significantly differentially expressed between the inoculated accessions PI272560 and 36554 on the basis of tags per million (tpm, p < 0.05) and which were differentially expressed between the non-inoculated and inoculated variant of at least one of the accessions (p < 0.05, log2 fold change > 1). Specifications outside the columns show the p-value of the expression differences between the inoculated and the non-inoculated variant of the particular accession. Asterisks and specifications above columns show significant higher values of tags per million (tpm) in the inoculated accession PI272560, asterisks below columns in the inoculated accession 36554. Non-significant differences are abbreviated with “n.s.” [file Image_1.TIF]

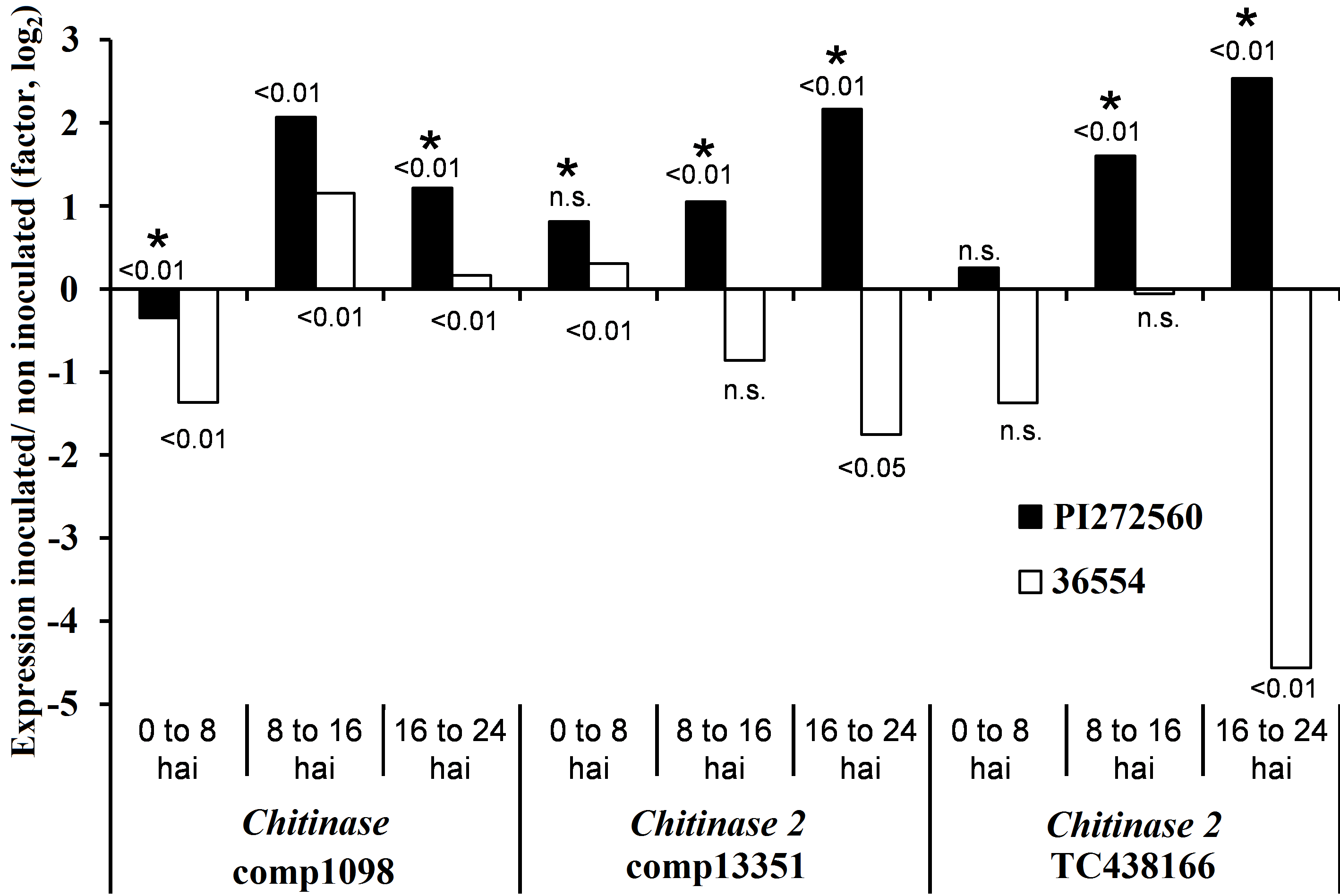

Supplement: FIGURE S2 — Expression of genes matching the GO-term defining “chitinase activity” (GO:0004568) which were identified in at least one time segment as significantly differentially expressed between the inoculated accessions PI272560 and 36554 on the basis of tags per million (tpm, p < 0.05) and which were differentially expressed between the non-inoculated and inoculated variant of at least one of the accessions (p < 0.05, log2 fold change > 1). Specifications outside the columns show the p-value of the expression differences between the inoculated and the non-inoculated variant of the particular accession. Asterisks and specifications above columns show significant higher values of tags per million (tpm) in the inoculated accession PI272560, asterisks below columns in the inoculated accession 36554. Non-significant differences are abbreviated with “n.s.”. [file Image_2.TIF]

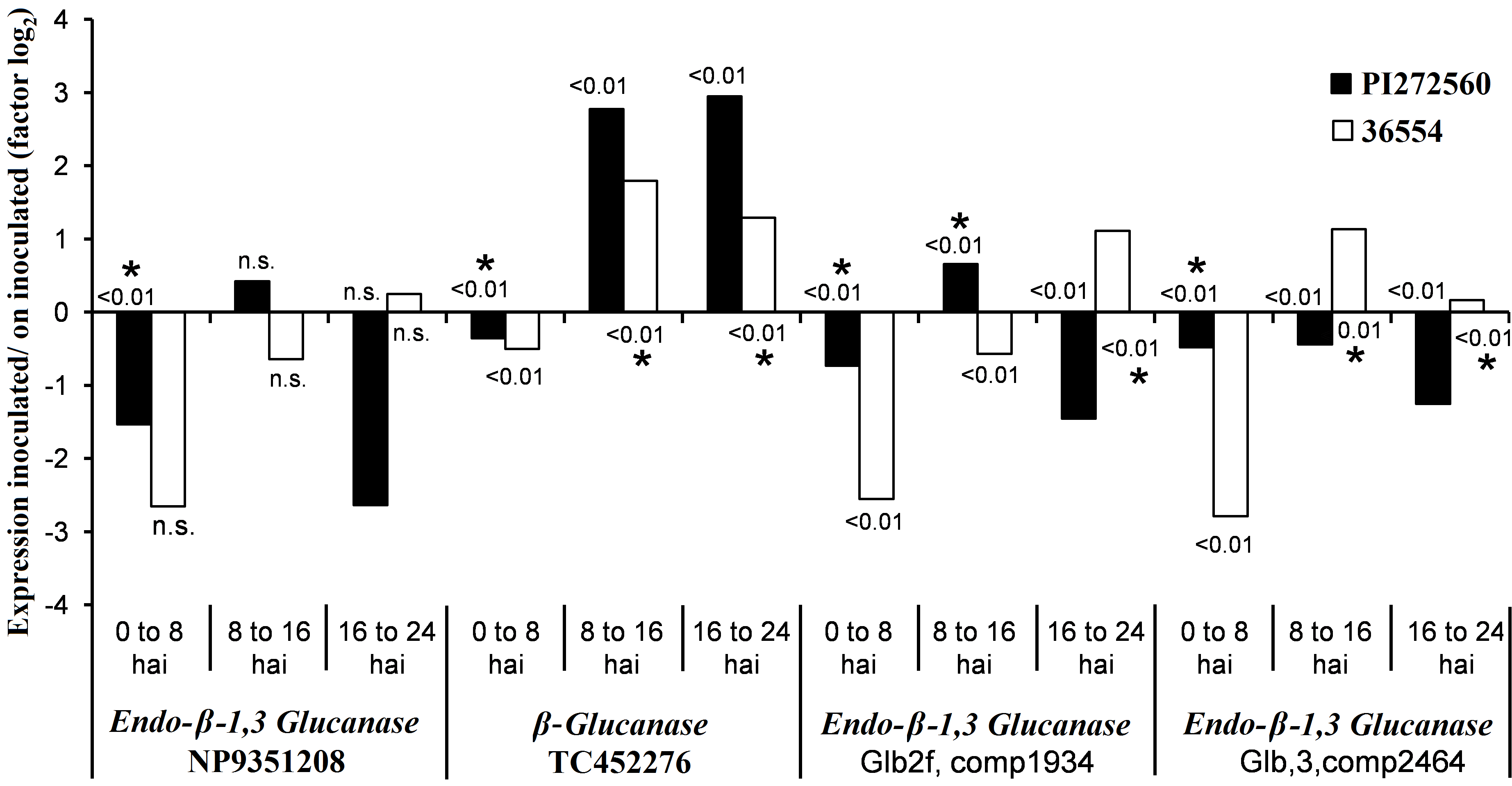

Supplement: FIGURE S3 — Expression of genes matching the GO-term “carbohydrate metabolic process” (GO:0005975) which were identified in at least one time segment as significantly differentially expressed between the inoculated accessions PI272560 and 36554 on the basis of tags per million (tpm, p < 0.05) and which were differentially expressed between the non-inoculated and inoculated variant of at least one of the accessions (p < 0.05, log2 fold change > 1). Specifications outside the columns show the p-value of the expression differences between the inoculated and the non-inoculated variant of the particular accession. Asterisks and specifications above columns show significant higher values of tpm in the inoculated accession PI272560, asterisks below columns in the inoculated accession 36554. Non-significant differences are abbreviated with “n.s.”. [file Image_3.TIF]

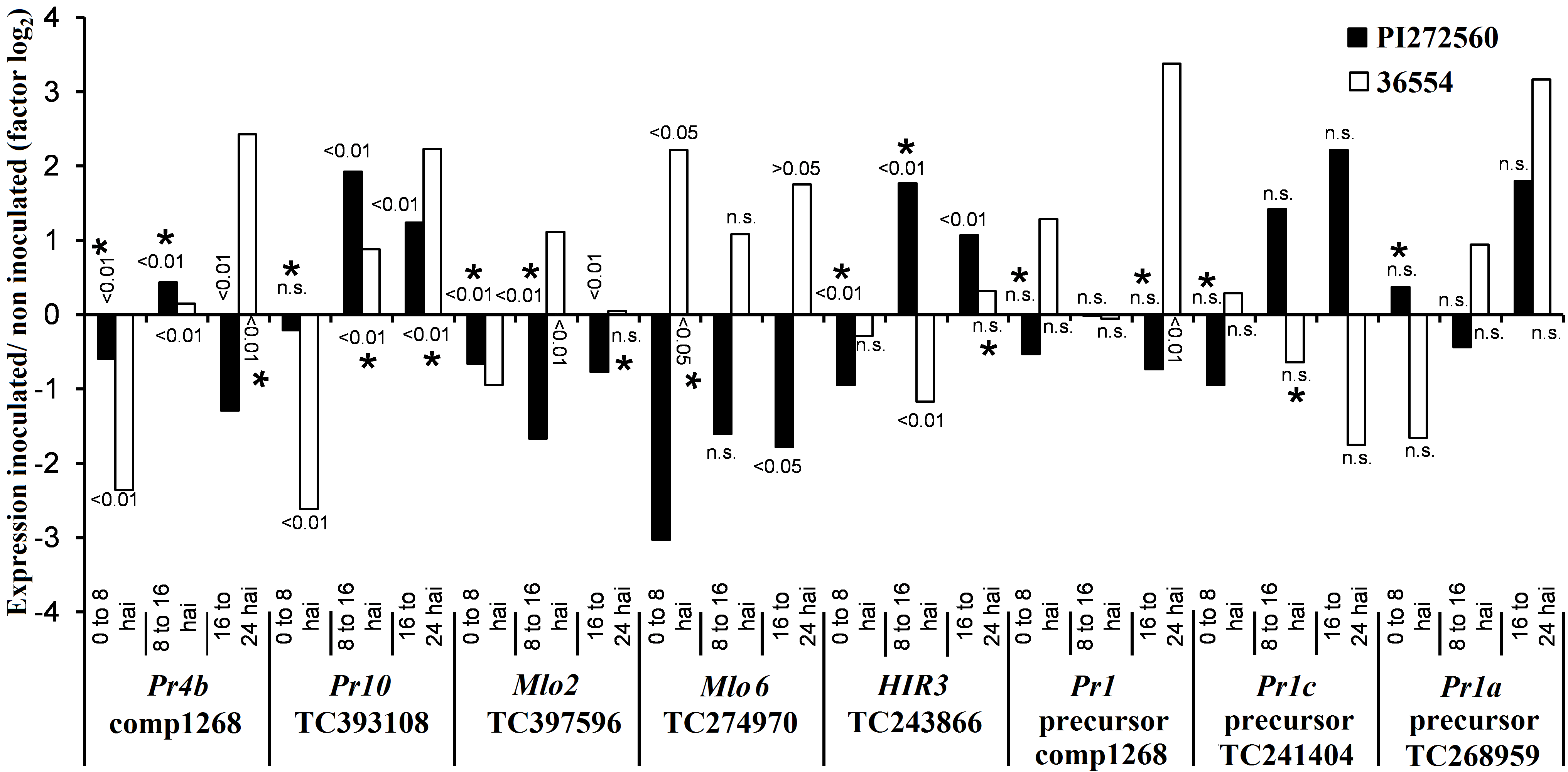

Supplement: FIGURE S4 — Expression of genes matching the GO-terms “defense response” (GO:0006952) “defense response to fungus” (GO:0050832) and “plant-type hypersensitive response” (GO:0009626) which were identified in at least one time segment as significantly differentially expressed between the inoculated accessions PI272560 and 36554 on the basis of tags per million (tpm, p < 0.05) and which were differentially expressed between the non-inoculated and inoculated variant of at least one of the accessions (p < 0.05, log2 fold change > 1). Specifications outside the columns show the p-value of the expression differences between the inoculated and the non-inoculated variant of the particular accession. Asterisks and specifications above columns show significant higher values of tpm in the inoculated accession PI272560, asterisks below columns in the inoculated accession 36554. Non-significant differences are abbreviated with “n.s.”, the Hypersensitive-induced reaction protein 3 with “HIR3.” [file Image_4.TIF]
